# *In vivo* pH measurement at the site of calcification in an octocoral

**DOI:** 10.1038/s41598-017-10348-4

**Published:** 2017-09-11

**Authors:** C. Le Goff, E. Tambutté, A. A. Venn, N. Techer, D. Allemand, S. Tambutté

**Affiliations:** Marine Biology Department, Centre Scientifique de Monaco, 8 Quai Antoine Ier, MC-98000 Monaco, Monaco

## Abstract

Calcareous octocorals are ecologically important calcifiers, but little is known about their biomineralization physiology, relative to scleractinian corals. Many marine calcifiers promote calcification by up-regulating pH at calcification sites against the surrounding seawater. Here, we investigated pH in the red octocoral *Corallium rubrum* which forms sclerites and an axial skeleton. To achieve this, we cultured microcolonies on coverslips facilitating microscopy of calcification sites of sclerites and axial skeleton. Initially we conducted extensive characterisation of the structural arrangement of biominerals and calcifying cells in context with other tissues, and then measured pH by live tissue imaging. Our results reveal that developing sclerites are enveloped by two scleroblasts and an extracellular calcifying medium of pH 7.97 ± 0.15. Similarly, axial skeleton crystals are surrounded by cells and a calcifying medium of pH 7.89 ± 0.09. In both cases, calcifying media are more alkaline compared to calcifying cells and fluids in gastrovascular canals, but importantly they are not pH up-regulated with respect to the surrounding seawater, contrary to what is observed in scleractinians. This points to a potential vulnerability of this species to decrease in seawater pH and is consistent with reports that red coral calcification is sensitive to ocean acidification.

## Introduction

Molecular phylogenetic research suggests that cnidarians originated 741 million years ago (Ma) and that the divergence between Hexacorallia and Octocorallia occurred prior to the Cambrian (543 Ma)^1^. It is not clear whether these two subclasses acquired the ability to calcify before or following their divergence. Hexacorallia include the “stony” reef-building corals, belonging to the order scleractinia, which produce skeletons made of calcium carbonate (CaCO_3_) precipitated as the polymorph of aragonite. Octocorallia resemble stony corals in general appearance, but their skeletons are different in both morphology and composition. Members of the Octocorallia produce two skeletal structures: an axial skeleton and sclerites. Depending on the species, the axial skeleton may be non-calcareous and proteinaceous in nature, or calcareous and formed from the aggregation of CaCO_3_ sclerites. In the case of the genus *Corallium*, which includes the Mediterranean red coral, some sclerites are incorporated into the axial skeleton at the apex of branches, but most of the calcareous sclerites remain embedded in the tissues and the calcareous axial skeleton is formed separately by different cells. Both axial skeleton and sclerites are composed of polymorph high-magnesium calcite^2^.

Determining and comparing the strategies that Hexacorallia and Octocorallia employ to produce their calcium carbonate biominerals holds the potential to provide novel insight into both mechanistic and evolutionary aspects of calcification. One question of particular interest is how members of both groups control physio-chemical parameters at internal sites of calcification. Among these parameters, pH is known to play a key role in calcium carbonate formation. Indeed it has been shown in scleractinian corals that pH at the site of calcification is “up-regulated” to values higher than the surrounding seawater. This pH up-regulation is under biological control and favors the formation of carbonate from other forms of dissolved inorganic carbon, leading to an increase in the saturation state of aragonite, favoring the calcification reaction Ca^2+^  + CO_3_^2−^ → CaCO_3_^3^. It has been speculated that the capacity of scleractinian corals to up-regulate pH at the site of calcification determines their response to ocean acidification caused by rising anthropogenic CO_2_^4, 5^. Information on pH up-regulation in corals comes either from indirect measurements using the boron isotope composition of skeletons^6–8^, or direct measurements either by the insertion of invasive microelectrodes through the tissues^4, 9–11^ or application of non-invasive pH-sensitive fluorescent dye, such as the SNARF-1 (carboxyseminaphthorhodafluor-1)^7, 12, 13^. Currently, almost all available information on pH regulation at the site of calcification in coral comes from studies on Hexacorallians, specifically scleractinians. There is no information on pH at the site of calcification in Octocorallia, apart from a boron isotope measurement of skeletal material from a deep sea *Corallium* sp. Interestingly, this measurement suggests that pH up-regulation at the site of calcification in this Octocorallian species is minimal or absent^14^. Furthermore, there is relatively little histological information about the site of calcification in octocorals i.e. about the relationship between the calcifying tissues and the two biominerals (axial skeleton and sclerites) that members of this group produce. This knowledge-gap exists in part due to the difficulties of gaining direct access to the calcifying tissues that lie under other tissue layers.

Recent advances in studying the calcifying tissues and pH regulation at the site of calcification in scleractinian corals have been facilitated by the use of live tissue imaging on coral microcolonies cultivated on glass slides or coverslips. Indeed, this method of culture, initially developed for ultrastructural studies^15^ has more recently been used in various studies (Table S1) including pH measurements by live tissue imaging with fluorescent dyes and confocal microscopy^5, 7, 12, 13, 16^. The use of live tissue imaging of microcolonies on glass slides has allowed direct observations and physiological measurements of the skeleton-tissue interface where calcification occurs. In the current study, we adapted this method for an Octocorallia species in order to obtain information on pH regulation at the site of calcification. We studied the precious Mediterranean coral *Corallium rubrum* which is widely known due to its use in jewelry and art objects for centuries^17, 18^. In the context of investigating the physiology driving biomineralization, *C. rubrum* offers an interesting comparison to scleractinians because it produces two distinct biominerals (an axial skeleton and sclerites) which differ by their size, morphology and location within the colony. Furthermore, both biominerals in *C. rubrum* are made of high-Mg calcite, in contrast to the aragonite skeleton found in scleractinians.

First we developed the coral culturing technique and characterized the biological material, in particular the histology of the microcolonies growing on glass coverslips. Then, in order to obtain structural information regarding the site of calcification, we investigated the interface between the tissues and the biominerals, together with the different steps involved in axial skeleton and sclerite formation. Finally we used the pH sensitive dye SNARF-1 and confocal microscopy to measure pH at the site of calcification of the two biominerals. Our results are discussed in the context of physiological regulation of biomineralization in scleractinian corals and octocorals.

## Results

### Organization of a microcolony

**Figure 1:**
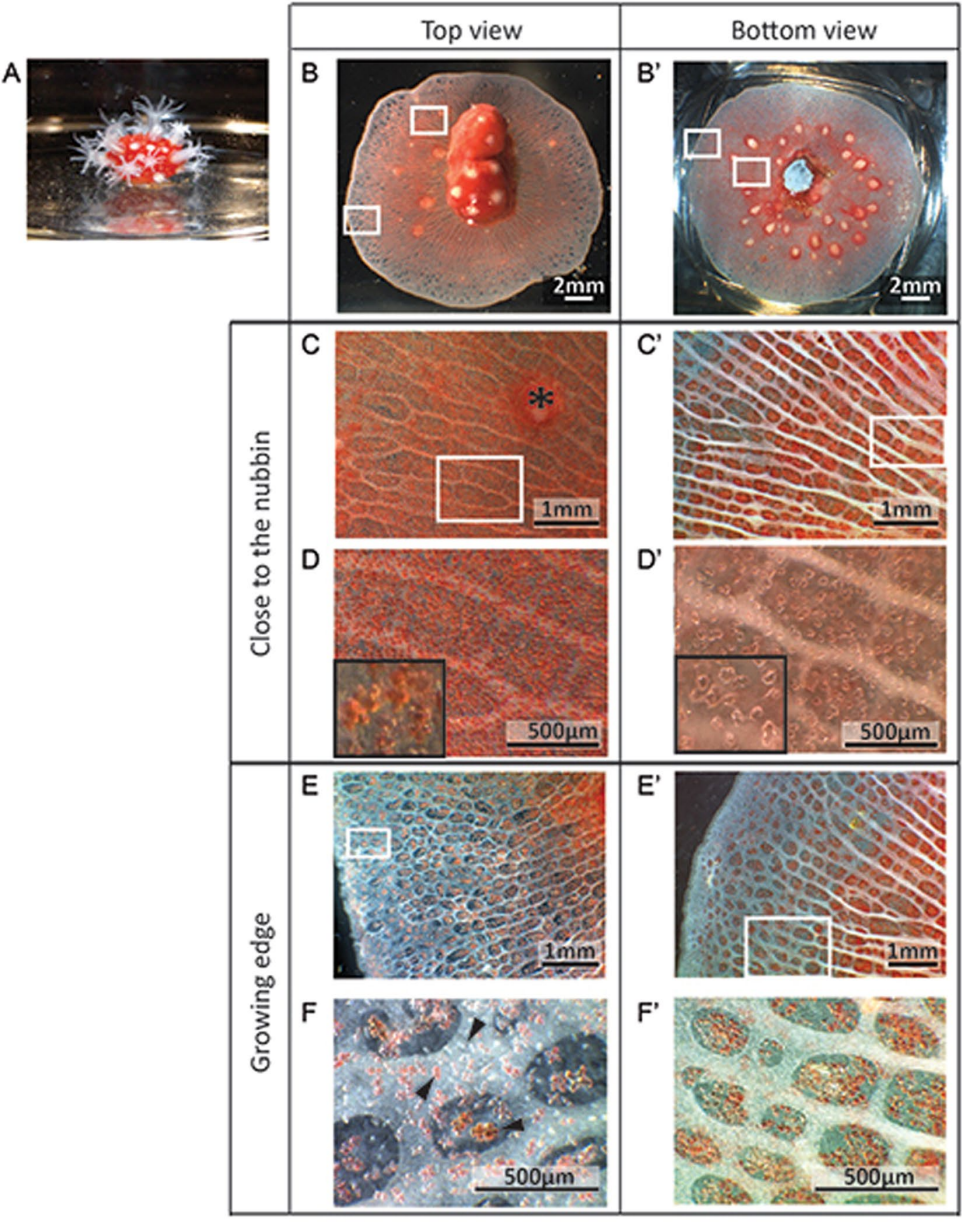
Tissue organization of a representative *C. rubrum* microcolony. (**A**) A branch apex cut and glued on a glass coverslip constituting a nubbin with open polyps. (**B**) and (**B’**) General views of microcolonies with the nubbin in the center of the preparation, tissues growing around and development of polyps. Boxes indicate two regions of interest: close to the nubbin and at the growing edge. (**B–F**) Views from above the microcolony. (**B’–F’**). Views from underneath. (**C**) and (**C’**) Are enlargements of tissue close to the nubbin, the coenenchyme is red colored and contains numerous parallel white gastrodermal canals. The asterisk indicates a retracted polyp. (**D**) Enlargement of (**C**) showing the high density of red sclerites. (**D’**) Enlargement of (**C’**) showing the mineralized sheets deposited on the coverslip under tissues. (**E**) and (**E’**) Are enlargements of tissue at the growing edge showing that canals are not fully developed at the extremities and sclerites are less dense. (**F**) Enlargement of (**E**) with black arrowheads showing white, pink/orange and red sclerites. (**F’**) Enlargement of (**E’**) showing the absence of crystals deposited on the coverslip at the growing edge.

Lateral extension of tissue began in all directions at three weeks after gluing cut apexes on glass coverslips (Fig. 1A), and resulted in new skeleton deposited on the glass slide after four months (Fig. 1B). Mean lateral linear extension rate of microcolonies were 64.72 ± 9.21 µm per day (n = 17). The anatomy of microcolonies was initially characterized from above by upright microscope (Fig. 1B–F). The central zone corresponding to the initial coral fragment glued on the coverslip is referred to as the “nubbin” whereas the most distant zone from the nubbin corresponds to the “growing edge”. The apparent red color of the tissue is due to the presence of colored sclerites (Fig. 1C,D). At the growing edge (Fig. 1F), sclerites display different sizes and colors (white, orange/pink and red). Relation between the size and the color was investigated by immersing a one-year-old microcolony upside down in dilute sodium hypochlorite to dissolve soft tissues. Color and size of isolated sclerites (n = 163) after sedimentation and localized at the growing edge were determined. Statistical analysis showed a significant difference between the three groups: White sclerites (32.0 ± 9.1 µm, n = 60), orange/pink (56.2 ± 8.5 µm, n = 43) and red (61.4 ± 5.5 µm, n = 60) sclerites; suggesting that the red coloration is acquired during the sclerite growth (one-way ANOVA, F(2,160) = 233.03, P < 0,001). The cœnenchyme, i.e the tissue between polyps is composed of more or less parallel endodermal canals (which appear white) linked together by anastomosis, running from the nubbin (Fig. 1C) to the growing edge (Fig. 1E) where they are not fully developed. These canals correspond to the gastrodermal system and communicate with the gastrovascular cavities of polyps. Observations of the bottom view of the microcolonies (Fig. 1B’–F’) were also made by turning microcolonies upside down. Close to the nubbin (Fig. 1C’), canals are more developed than at the growing edge (Fig. 1E’). Crystals are deposited on the coverslip under tissues to form mineralized sheets (Fig. 1D’), except at the growing edge where crystal deposition is absent (Fig. 1F’). Sclerites are located above the canals, in the upper part of the cœnenchyme.

**Figure 2:**
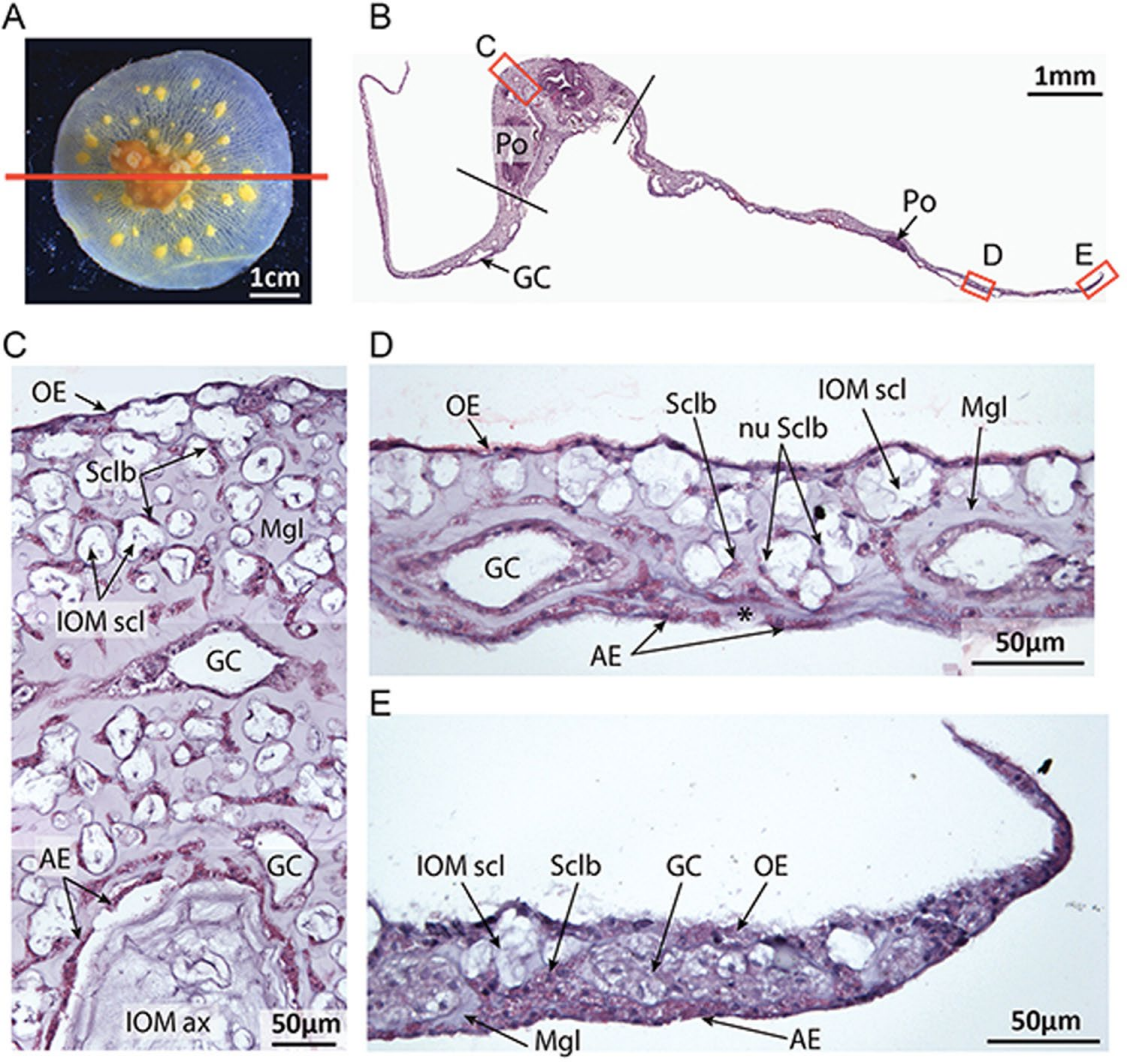
Sagittal section of a decalcified microcolony stained with hematoxylin and eosin. (**A**) Photo of a decalcified microcolony used for sagittal section as indicated by the red line. (**B**) Reconstruction of the general view of the stained sagittal section with hematoxylin and eosin. The nubbin is localized between the two black lines. The adjacent parts correspond to the tissue growing on the coverslip (Scale: 1 mm). (**C**) Histology at the nubbin**’**s apex (Scale: 50 μm). (**D**) Histology of the tissue growing on the coverslip. The asterisk indicates secreted IOM by the aboral ectoderm (Scale: 50 μm). (**E**) Histology of the tissue growing at the growing edge on the coverslip (Scale: 50 μm). Po: Polyp; GC: Gastrodermal canals; OE: Oral ectoderm; AE: Aboral ectoderm; Mgl: Mesoglea; Sclb: Scleroblasts; nu Sclb: Scleroblasts nuclei; IOM scl: Insoluble organic matrix of sclerites; IOM ax: Insoluble organic matrix of the axial skeleton.

A sagittal section was cut in the middle of a fixed and decalcified microcolony (Fig. 2A), and stained with hematoxylin/eosin (Fig. 2B) in order to gain information on the histology of the microcolony. In the nubbin (Fig. 2C), the cœnenchyme is composed of two epithelia separated by the mesoglea: the oral ectoderm facing the seawater, and the aboral ectoderm responsible of axial skeleton formation. Between the two ectoderms, the cœnenchyme consists of mesoglea, a gastrodermal canal network (multilayer organized), and many scleroblasts (*i.e*. responsible for sclerite formation) which surround the insoluble organic matrix (IOM) of sclerites (called “sclerites ghosts”) that remains after decalcification. These sclerite ghosts are present throughout the cœnenchyme, but in higher density under the oral ectoderm. The nubbin (which was formally a branch apex) thus displays the typical histology of a *C. rubrum* branch^19, 20^. Figure 2D,E shows a sagittal section cut on the tissue growing on the glass coverslip, respectively 6 and 7 mm distant from the nubbin. In both cases, the cœnenchyme is thinner than in the nubbin. In Fig. 2D, the gastrodermal canals are distributed in a monolayer and sclerites are present either throughout the mesoglea in the absence of canals, or above canals in the upper part of the cœnenchyme. Ghosts of sclerites corresponding to the IOM are surrounded by one or several scleroblasts as can been seen with the dark staining of nuclei. The aboral ectoderm appears as a cell monolayer, and is as thin as the oral ectoderm. Although the IOM of sclerites was well preserved, the IOM secreted by the aboral ectoderm on the coverslip is lost during the preparation and only a small fragment is visible (indicated by an asterisk). With closer proximity to the growing edge (Fig. 2E), the thinner the mesoglea and the less the gastrodermal canals are developed. The oral and aboral ectoderms are still distinguishable except at the proximal edge of the growing tissue, where they are joined with a disorganized aggregation of cells as revealed by several dark cell nuclei (Fig. 2E).

### Biominerals deposited on the coverslip

**Figure 3:**
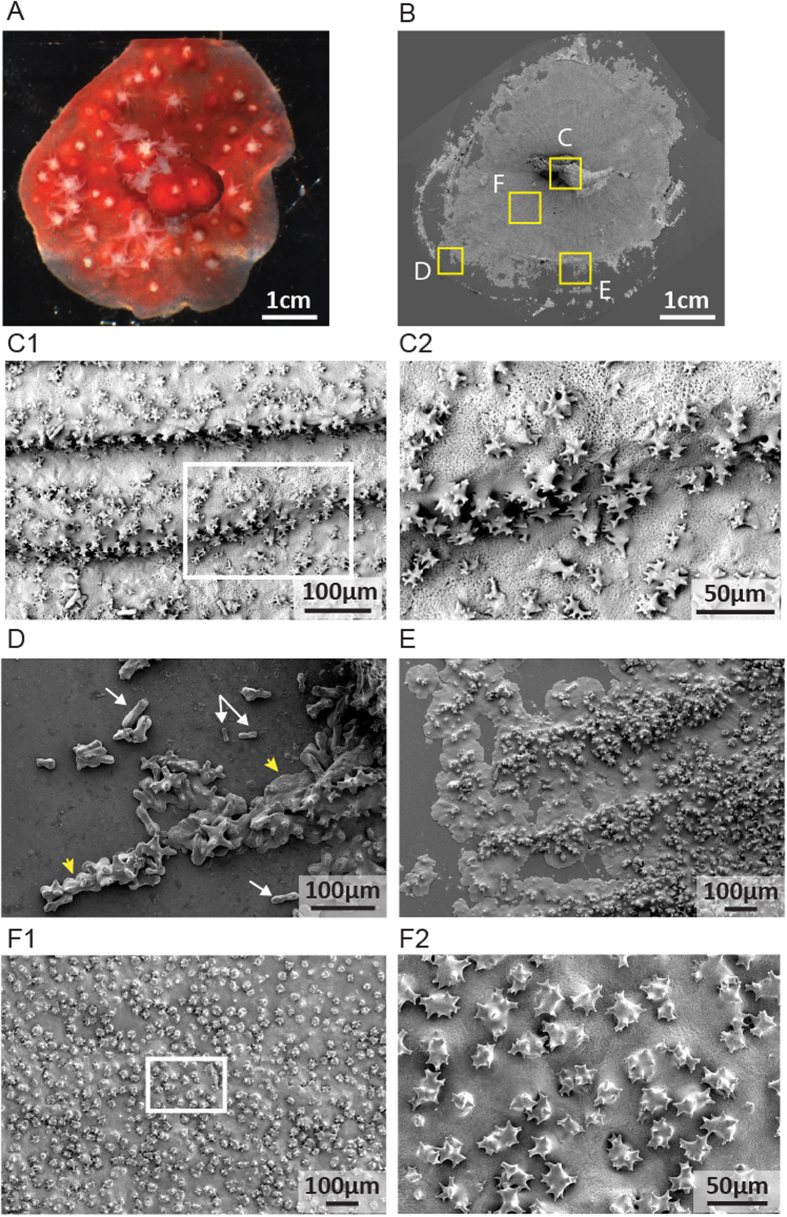
Microstructure of the newly formed axial skeleton in a *C. rubrum* microcolony processed for SEM. White boxes indicate enlargements. (**A**) Microcolony before removal of soft tissues. (**B**) General view of crystals deposited on the coverslip. Yellow boxes indicate four regions of interest. (**C1,C2**) Organization of the nubbin**’**s axial skeleton. (**D**) First stage of skeleton formation with dumbbell-shaped crystals deposited (white arrows), then embedded in a mineral sheet (yellow arrows). (**E**) Later stage of skeleton formation with mineral sheets covered with microprotuberances. (**F1,F2**) More advanced stage of skeleton formation with a thick skeleton covered with a high density of microprotuberances.

Removal of soft tissues by dissolving the microcolony in NaOH allows observation of different stages of biomineral deposition leading to skeleton formation on the glass coverslip (Fig. 3). Zone C represents the axial skeleton of the nubbin. Zones D to F, from the growing edge to the nubbin, represent respectively the earlier and the later calcification steps to form the skeleton. In zone D, the most distant zone from the nubbin, crystals with a dumbbell shape are first deposited on the coverslip (Fig. 3D, white arrows), and are then embedded in a mineral sheet (Fig. 3D, yellow arrowheads). Observation of microprotuberance formation on scanning electron images combined with immunolabelling with an antibody against soluble organic matrix of axial skeleton show that these dumbbell-shaped crystals give rise to microprotuberances (Fig. S2). Going from the growing edge to the nubbin, these mineral sheets enlarge, join together to form larger plaques where microprotuberances are observed (Fig. 3E). With closer proximity to the nubbin, the thicker the plaques and the higher the density of microprotuberances (Fig. 3F1–F2) which finally look like the axial skeleton (Fig. 3C1–C2).

### Interfaces between calcifying cells and biominerals

Different imaging techniques (optical, confocal, transmission and scanning electron microscopy) were used to study the spatial relationship between the calcifying cells and the biominerals on whole microcolonies.

#### Growing environment of skeleton biominerals

The incubation of a microcolony at different time intervals in seawater containing the cell impermeant fluorescent calcium-binding dye calcein allows us to confirm that the crystals were actively growing (Fig. S3A). The calcein labelling was also used in combination with Phalloidin (for cytoskeleton labelling) and DAPI (for nuclei labelling) to investigate the interface between the crystals and the surrounding cells. It can be observed that a mineralized growing sheet is surrounded by more than one cell. Indeed many cells are in close contact with it (Fig. S3B). In this experiment with Phalloidin, no primary crystals were observed.

#### Growing environment of sclerites biominerals

**Figure 4:**
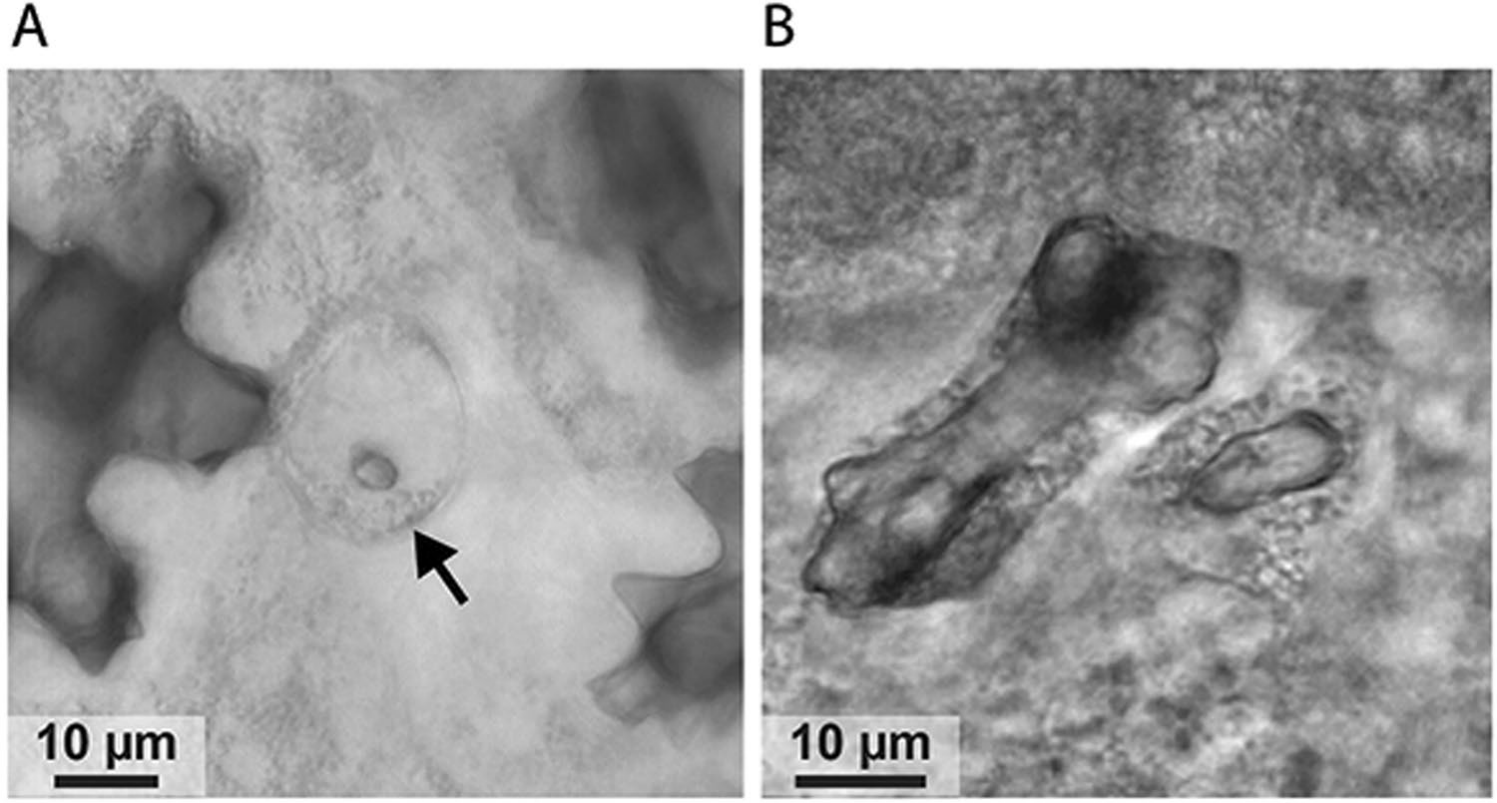
Transmitted lights images of *in vivo* observations of sclerites. (**A**) Hypothetical intracellular stage of sclerite initiation indicated by the black arrow. (**B**) Two growing sclerites at different stages of growth: (i) a larger sclerite in the terminal phase of growth (ii) a smaller sclerite prior to formation of protuberances.

**Figure 5:**
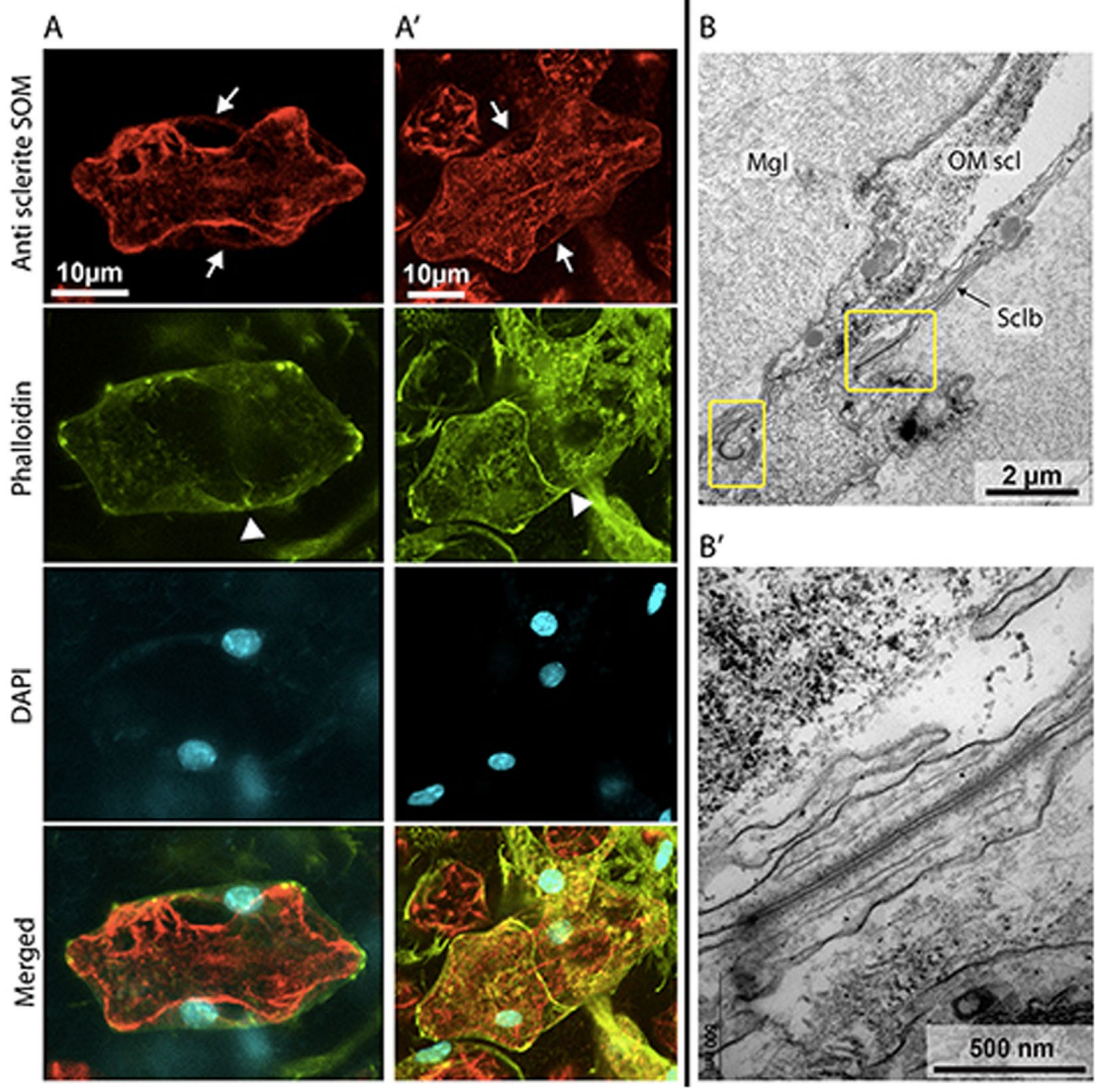
Observation of growing sclerites and their cellular environment by confocal microscopy and TEM. (**A-A’**) Confocal images of growing sclerites labeled with anti sclerite SOM (red), and scleroblasts labeled with Phalloidin (actin, green) and DAPI (nuclei, blue). Arrows suggest the secretion of OM at the level of scleroblasts membranes. Arrowheads show junction between scleroblasts. The merged images show the cellular environment of growing sclerites, surrounded by two scleroblasts. (**A**) Observation of a section along the optical axis Z in a sclerite. (**A’**) Maximum intensity projection (MIP) of a z-stack of multiple optical sections with a 0.2 µm step between them. (**B-B’**) TEM images of a transversal section in a *C. rubrum* microcolony showing septate junctions between two scleroblasts. (**B**) Sclerite in the mesoglea surrounded by two scleroblasts. Yellow boxes indicate the presence of two septate junctions. (**B’**) Enlargement showing a septate junction between two scleroblasts. Mgl: Mesoglea; OM scl: Organic matrix of sclerite; Sclb: Scleroblasts.

Occasional *in vivo* observations of apparently mineralized structures surrounded by a cell in tissue growing on the coverslip suggest an initialization of sclerite calcification in a primary scleroblast (Fig. 4A_arrow). Growing sclerites were also observed (Fig. 4B), but these observations do not allow determination of the number of cells and nuclei around these biominerals. Further observations were thus performed on microcolonies growing on the coverslip after fixation and decalcification. The tissues were peeled off from the coverslip, cut into pieces and stained with Phalloidin, DAPI and an antibody against soluble organic matrix of sclerites (anti-sclerite SOM). The anti-sclerite SOM was used since it labels not only the SOM but also the insoluble organic matrix (IOM) of the sclerites which remains after decalcification of the tissues, due to the presence of common epitopes recognized by the antibody in both organic fractions^21^. Ghosts of sclerites of different size were observed, the smaller corresponding to growing sclerites (<50 µm) for which protuberances are not fully formed, and the bigger corresponding to mature sclerites for which protuberances are totally formed. Repeated observations confirmed that two nuclei can be observed around one growing sclerite, showing that one sclerite is surrounded by two scleroblasts (Fig. 5A-A’_DAPI). Moreover, Phalloidin labelling highlights a boundary between two scleroblasts (Fig. 5A-A’_Phalloidin, arrowheads). These observations on optical sections were confirmed by the maximum intensity projection (Fig. 5A-A’_Merged) that allows the complete visualization of the growing sclerite environment. Observations by transmission electron microscopy highlighted the presence of septate junctions between plasmic membranes of two scleroblasts (Fig. 5B-B’). Scleroblasts form a delimited medium with cellular membranes stretched around the protuberances of sclerites. Colocalization of Phalloidin with anti-sclerite SOM (Fig. 5A-A’_Anti sclerite SOM, white arrows) shows that organic matrix is secreted at these locations, and is indicative of sclerite active growth. Mature sclerites were also observed in the mesoglea. In these cases, the cellular environment is different from a growing sclerite with a cell network passing between protuberances (Fig. S4). Cells are in close contact with sclerites protuberances but do not surround nor envelop them.

### pH measurements

#### pHe

**Figure 6:**
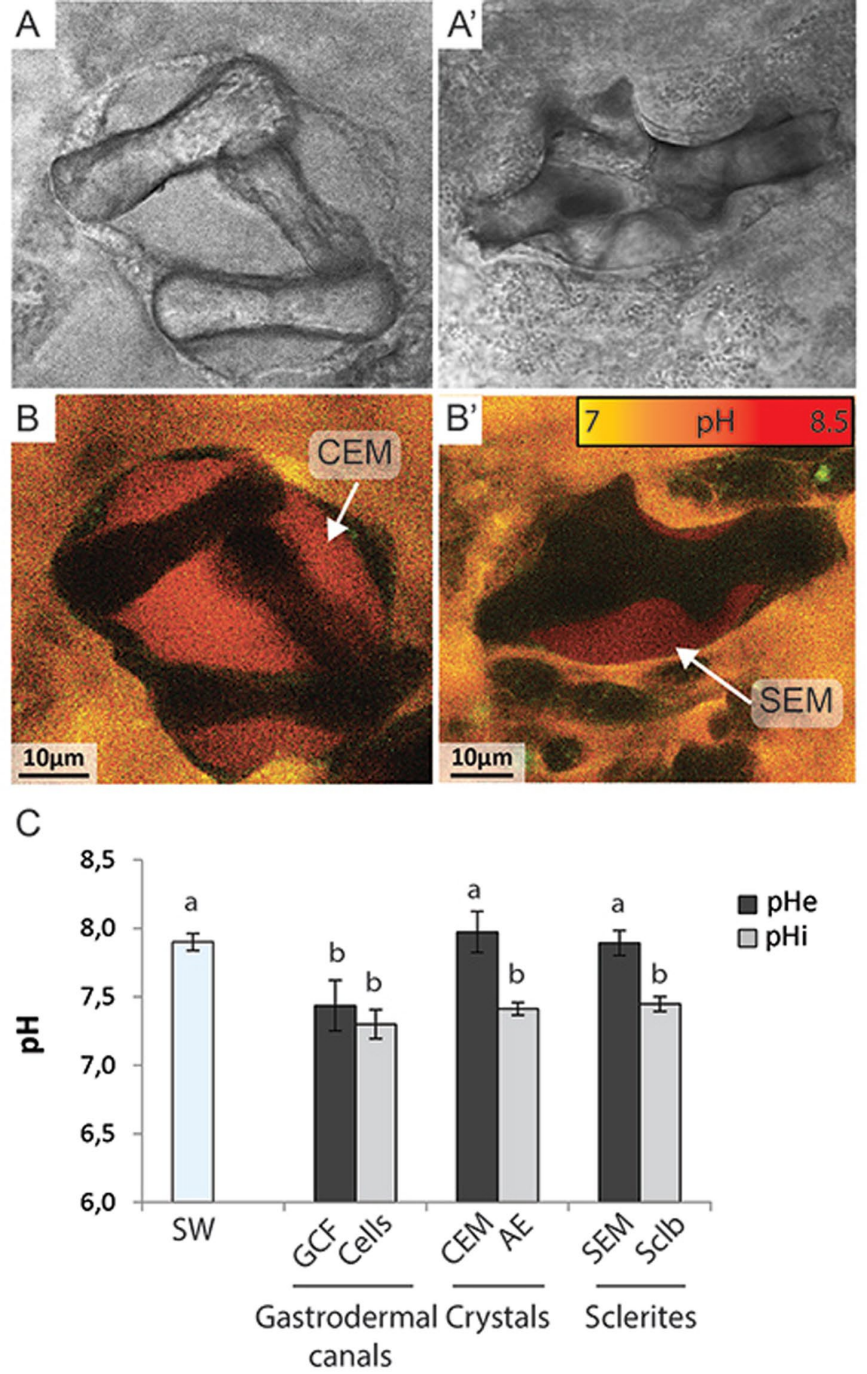
Confocal images of SNARF-1 labeling of the calcifying medium of biominerals and pH measurements in different intra- and extracellular compartments. Transmitted light images of dumbbell-shaped crystals deposited on the coverslip (**A**) and a growing sclerite in the mesoglea (**A’**). (**B-B’**) Fluorescence images of SNARF-1 labeling showing the presence of an extracellular calcifying medium around biominerals. CEM: Cristal Extracellular Medium; SEM: Sclerite Extracellular Medium. (**C**) *In vivo* pH measurements obtained in cells and calcifying extracellular medium of *C. rubrum* microcolonies. Data are compared to the seawater pH (SW) surrounding microcolonies. Data are mean per microcolony ± standard deviation. SW: Seawater (n = 7); GCF: Fluid of gastrodermal canals (n = 4); CEM: Crystals extracellular medium (n = 5); AE: Aboral epithelium (n = 4); SEM: Sclerites extracellular medium (n = 4); Sclb: Scleroblasts (n = 7). Letters indicate mean pH values significantly different (one-way ANOVA with Leven**’**s post-hoc).

Live microcolonies growing on coverslips incubated with the pH sensitive extracellular probe, SNARF-1, were observed with an inverted confocal microscope in order to visualize the extracellular calcifying medium surrounding the biominerals and to measure its pH. Despite repeated observations over several microcolonies, the extracellular calcifying medium was never observed around the mineralized sheets but was successfully observed between the aboral ectoderm and the crystals deposited on the glass coverslip (Fig. 6A,B). These crystals correspond to the dumbbell-shaped crystals observed in Fig. 3D which give rise to microprotuberances. The extracellular calcifying medium was also observed between the sclerites and the scleroblasts (Fig. 6A’,B’). pH values of 7.89 ± 0.09 and 7.97 ± 0.15 were respectively measured in the extracellular calcifying medium of crystals and sclerites (Fig. 6C). On the same microcolonies, pHe measurements were also obtained for the fluid flowing in the gastrodermal canals (7.44 ± 0.18) and for the seawater surrounding the microcolony (7.90 ± 0.06) at 100 µm from the sample, at the beginning and at the end of incubation (Fig. 6C).

#### pHi

pHi measurements were also performed in living microcolonies with the same protocol as described for pHe measurements except that the pH sensitive fluorescent dye used was SNARF-1-AM which is cell permeant. pHi was determined for endodermal cells forming the gastrodermal canals (7.30 ± 0.11), for scleroblasts (7.45 ± 0.05) and for the calcifying cells surrounding the crystals (7.41 ± 0.05) (Fig. 6C). Statistical analysis of pHe and pHi data (one-way ANOVA, F(6,30) = 41,31, P < 0,001) revealed that pHe of calcifying medium of the two types of biominerals is not significantly different from the seawater pH. However, pHe of these calcifying media is significantly higher than the pH of the fluid circulating in the gastrodermal canals. Moreover, there are no significant differences between pHi values measured in the different cellular types. It was also found that pHi of endodermal cells constituting gastrodermal canals is not significantly different from pHe of fluid circulating in gastrodermal canals. However, pHi of calcifying cells is significantly lower than pHe in extracellular calcifying medium.

## Discussion

We developed the preparation of microcolonies growing on glass coverslips for the Octocorallian *Corallium rubrum* in order to facilitate insight into the pH regulation at the site of calcification of this species and compare it to the results obtained for scleractinian corals. By different microscopic techniques we first characterized the anatomy and histology of the preparation, as well as the site of calcification where sclerites and crystals form and grow. This part of the work was a prerequisite step for the subsequent use of microcolonies for physiological measurements of pH at the site of calcification.

As shown previously for scleractinian corals, the use of microcolonies growing on glass coverslips is useful in studying early calcification processes occurring in *C. rubrum*. Indeed different stages of growth and calcification are observable in time and space, from the initial stages at the growing edge to the formation of the skeleton close to the nubbin. As observed for scleractinian corals, one of the advantages of this technique is that the microcolonies growth rate is higher than for branching colonies. Indeed, the mean growth rate obtained after four months on the coverslip was 64.72 ± 9.21 µm per day corresponding to about 25 mm per year for the tissue extension on the coverslip compared to the 0.20–0.35 mm per year for the growth rate in diameter^22–25^ or the 1–5 mm per year for the axial growth rate^23, 26–28^ of a colony branch. This difference of growth rate is probably due to a favorable substrate, the glass coverslip, which allows the continuous growth of the microcolony.

Compared to scleractinian corals, the limitations of the technique for *C. rubrum* are: (1) the low percentage of successful preparations since some nubbins never attach to the glass coverslip, (one successful nubbin for 5 failures); (2) the low growth rate of 64.72 ± 9.21 µm per day corresponding to about 2 mm per month compared to the 6–7 mm per month for scleractinian corals (Personal observation of the authors) and (3) the long term period of time (at least four months) before observations can be made due to the low growth rate compared to the few weeks for scleractinian corals.

The anatomy and histology of a *C. rubrum* microcolony on a glass slide is highly similar to descriptions of a branching colony^19^ with an oral ectoderm facing seawater, a mesoglea with scleroblasts containing sclerites, endodermal cells forming the gastrovascular canals, aboral ectodermal calcifying cells and the axial skeleton. However the difference and advantage compared to a branching colony is that the different steps of formation of both of cell layers and the two biominerals can be observed from the growing edge to the nubbin.

Concerning the axial skeleton, the pattern of formation consists in (1) the formation of dumbbell-shaped crystals on the coverslip which then give rise to microprotuberances, (2) the production of biomineral layers that expand, (3) followed by the thickening of the mineral sheets covered with microprotuberances, with the formation of longitudinal crenulations on the surface of the skeleton close to the nubbin. Concerning the formation of sclerites, our results suggest that the first step takes place intracellularly in a primary scleroblast. Then, observations from a previous work show that in *C. rubrum*, nearly mature sclerites are surrounded by several scleroblasts in contact by cytoplasmic extensions similar to pseudopodia^20^. However, the number of scleroblasts involved has never been determined until our study in which we have observed that sclerites continue to grow in an extracellular medium formed by two secondary scleroblasts that are attached together by septate junctions. Finally, mature sclerites were observed in the mesoglea where they have been extruded from secondary scleroblasts.

The process of sclerite formation in *C. rubrum* was compared to the data available in the literature for other Octocorallian species^29–37^ (summarized in Fig. S5). Even if all the steps of sclerite formation have not been characterized in these species, taken together these results suggest that, in Octocorallians, a transient intracellular step is followed by a longer process of extracellular growth and the release of mature sclerite in the mesoglea.

Our aim was to measure pH at the site of calcification of the sclerites and the axial skeleton both in the extracellular calcifying medium and in the calcifying cells. Additionally, our preparations allowed us to gain information on the pH in other compartments, specifically the endoderm cells and the non-calcifying gastrovascular canals. Our results are discussed in two parts: (i) pH values in the different cell types and extracellular compartments, (ii) regulation of pH in the calcifying medium and its implication for calcification.

Turning first to pH in the different cells and compartments, measurements of pHi in *C. rubrum* varied from a highest mean pHi of 7.45 in the scleroblasts to lowest mean pHi of 7.3 in the endoderm cells but there are no significant statistical differences between pHi values measured in the different cellular types. These values fall within the range measured previously for cnidarian cells^38–40^. Calcifying cells (scleroblasts and cells surrounding crystals) displayed a very similar pH to calcifying calicoblastic cells in scleractinians (e.g. pHi 7.4 in *S. pistillata*). Unlike scleractinians, in which endodermal cell pHi (pHi 7.13) is markedly lower than in the calcifying cells, in *C. rubrum* there was no significant difference between calcifying and endodermal pHi (note that the comparison is made with *S. pistillata* endodermal cells in which pHi has not been elevated by photosynthetic activity of intracellular symbionts). These pHi values (in the range of pH 7–7.5) favor the conversion of CO_2_ produced by cellular respiration into bicarbonate, the dominant form of dissolved inorganic carbon (DIC) in the cytoplasm of the cells. Even if the mechanisms of pHi regulation are largely uncharacterized for cnidarians, apart from a study on *Anemonia virdis* ^39^, in the case of *C. rubrum*, the conversion of CO_2_ into bicarbonate is likely to be kinetically favored by the presence of an intracellular and a secreted carbonic anhydrase which have been previously characterized in *C. rubrum* ^41^.

Although there is direct exchange of the fluid circulating in gastrodermal canals with seawater via the polyps, gastrodermal fluid pH was found to be much lower than the surrounding seawater pH. It is also interesting to note that it is lower than cœlenteron pH measurements in scleractinians (e.g. the coral *Galaxea fascicularis* measured under light (pH 8.19) and dark (pH 7.61) conditions using microeletrodes^42^. Reductions in gastrodermal canal pH in *C. rubrum* may occur potentially by secretion of protons into the gastrovascular fluid to promote digestive functions, or by the release of CO_2_ from endodermal cells that line the gastrodermal canals due to potentially high rates of cellular respiration in this cell type promoted by the availability of metabolites. The fact that pH of the gastrovascular fluid has a mean value of 7.44 which is not statistically different from the value of the intracellular pH of endodermal cells indicates that proton concentrations are in chemical equilibrium between the endoderm cells and adjacent gastrovascular fluid. However, since the transmembrane electrical potential is unknown in coral cells, it’s not yet possible to determine if protons are actively or passively distributed across the cell membrane.

In contrast to the low pH values in the gastrodermal fluid and its lack of proton gradient with endodermal cells, a steep pH gradient was found to occur with the calcifying cells (~pH 7.4) and the calcifying fluid (pH 7.89 for sclerites and pH 7.97 for crystals). To maintain such a gradient, cells have to actively remove protons from the extracellular calcifying fluid or to add base equivalent to the site of calcification. In scleractinian corals, a Ca^2+^-ATPase specifically localized in the calcifying cells is considered as a candidate for regulating pH at the site of calcification^43^. This process in scleractinian corals is thus energy dependent and further research will be necessary to determine if such a mechanism is also present in *Corallium rubrum*.

Up-regulating pH of the calcifying medium increases the concentration of carbonate ions and thus elevates the saturation state of CaCO_3_ (Ω_CaCO3_) relative to the surrounding seawater. As this process favors the calcification reaction, the setting up of an elevated saturation state at the site of calcification is considered to be a key step in the biomineralization process^44^. Indeed pH up-regulation of the calcifying medium has been observed (1) in light and dark conditions, (2) in symbiotic and non-symbiotic organisms, and (3) independently of calcitic or aragonitic biomineral production. This is the case, for scleractinian corals (Table S2), foraminifera^45–47^, coccolithophores^48^ and also calcareous green algae^49–51^. Contrary to these organisms, our data in *C. rubrum* do not show an up-regulation relative to the surrounding seawater. This result confirms the only available indirect measure obtained by the boron isotope technique on *Corallium* sp.^14^ and clearly shows that up-regulating pH of the calcifying medium compared to seawater is not an ubiquitous process among corals. Such a result could also be interpreted to mean that saturation states in the calcifying fluid are lower in *C. rubrum* than in scleractinian corals, which could explain why rates of calcification in *C. rubrum* are lower than in scleractinian corals.

From a mechanistic point of view, the similarity between the value of pH at the site of calcification and seawater pH (both for sclerites and axial skeleton) could suggest that the calcifying medium is in relation (equilibrated?) with external seawater through an exchange occurring between cells through the septate junctions. In this case two possibilities can be considered: (1) if the renewal rate of the calcifying medium by seawater is low and that DIC disequilibrium with the surrounding seawater occurs due to consumption of carbonate for calcification, then it’s necessary to regulate calcifying fluid pH in order to maintain it at the value close to pH 8; (2) if the renewal rate of the calcifying fluid with seawater is sufficiently high, then it’s not necessary to regulate pH to keep it close to seawater pH values. However it should be noted that similarity between seawater pH and pH at the site of calcification does not necessarily mean that the ionic composition at the site of calcification is the same as seawater. Further investigations are necessary to determine the extent to which the paracellular *versus* the transcellular pathway plays a role in determining the composition of the calcifying medium.

It is important to consider that pH is only one of the parameters that are likely to govern calcification rates and that other factors are likely to be involved. For example, the concentration of DIC in the calcifying medium will also determine saturation state. Supply of inorganic carbon to the calcifying medium is proposed to occur by transcellular processes in scleractinian corals^52, 53^, but this is yet to be described in *C. rubrum*. An alternative way to promote calcification is to enhance calcium concentrations by active supply through transcellular transport^43, 54^ or to increase the nucleation rate of calcium carbonate crystals in the presence of organic components^55^. Indeed in the case of the fish otolith, gradients of organic compounds are hypothesized to be the primary drivers of calcification, whereas pH is not^56^. Organic compounds have been characterized in *C. rubrum* ^19, 21^ but further research is needed to understand their role in calcification.

Finally, despite the fact that under control conditions *C. rubrum* does not up-regulate pH in the calcifying medium compared to seawater, another important issue will be to determine whether it is able or not to regulate pH under seawater acidification. Indeed it has been shown in scleractinian corals that this capacity is species-specific and explains, at least partly, the sensitivity/resistance of scleractinian corals to ocean acidification (OA)^4^. Moreover, it has been recently shown that sclerite and axial skeleton formation is affected by OA in *C. rubrum* ^57, 58^. Our findings suggest that these previous observations of alterations of CaCO_3_ skeletal structures could be explained by dissolution processes due to the absence of pH up-regulation at the site of calcification in acidified conditions. Further experiments designed to measure pH at the site of calcification under these conditions will help in understanding the mechanistics that is involved in the response of this coral to OA.

## Conclusions

In the present study we developed the culture of microcolonies of *Corallium rubrum* which allowed us to obtain, for the first time in an Octocorallian species, direct *in vivo* pH measurements in different cell types and at sites of calcification. Despite the fact that calcifying media are more alkaline compared to calcifying cells and fluids in gastrovascular canals, pH at sites of calcification are not up-regulated with respect to seawater, confirming interpretation of boron isotope data that pH up-regulation in *Corallium* sp. is weak or absent^14^. This lack of pH up-regulation is contrary to what is observed in scleractinian corals and further research is required to determine whether this trait extends to all calcifying Octocorallians. If this were found to be the case, then it would suggest that octocorals have evolved different physiological mechanisms of calcification from scleractinians and other marine calcifiers that pH up-regulate. Turning to the issue of ocean acidification, our results suggest that the reported sensitivity of *C. rubrum* to decrease in seawater pH could be explained by the absence of pH up-regulation at site of calcification. Future experiments designed at determining whether *C. rubrum* is able or not to regulate pH at sites of calcification under seawater acidification will be necessary to confirm this hypothesis. More broadly, the current study lays the foundation for future research into physiological aspects of calcification in octocorals that is valuable for a better mechanistic understanding of biomineralization in corals and other marine calcifying organisms.

## Material and Methods

### Biological material and preparation of microcolonies

Experiments were performed on the Octocorallian *Corallium rubrum* (Supplementary Material). Microcolonies growing on glass coverslips were obtained from branches with a similar protocol to that used previously for *Stylophora pistillata* ^13, 15^. Apexes of branches were removed with a bone cutter in order to obtain fragments called “nubbins” which were glued to glass coverslips with epoxy resin mixture (Devcon). Nubbins on coverslips were maintained in aquariums in darkness for several months and allowed to grow laterally across coverslips in order to obtain microcolonies. Algae were removed once a week from the coverslips using a razorblade.

### Light microscopy for anatomy/histology of microcolonies

To obtain histological sections, microcolonies were fixed overnight in 4% paraformaldehyde in S22 buffer (450 mM NaCl, 10 mM KCl, 58 mM MgCl_2_, 10 mM CaCl_2_, 100 mM Hepes, pH 7.8) at 4 °C under gentle agitation. After fixation, samples were decalcified for one week using 0.25 M EDTA in calcium-free S22 under gentle agitation. Microcolonies were then gently peeled off the coverslip with a surgical blade before being dehydrated through a graded series of ethanol ending with a concentration of 100% and finally embedded in paraffin wax. 5 μm thick sagittal sections were cut through microcolonies. Deparaffinized sections were mounted on silane-coated glass slides and colored with hematoxylin/eosin (H-E) to stain nuclei and cytoplasm, respectively. Sections were observed under bright light with a polarizing microscope Leica DM750P.

### Transmission and Scanning Electron Microscopy for histology of microcolonies

Samples preparation, fixation and decalcification for Transmission Electron Microscopy (TEM) were performed according to protocols described in Tambutté *et al*.^59^. Briefly, microcolonies were fixed, decalcified, post-fixed and finally embedded in Epon resin (Supplementary Material). Observations of 1 µm thick sections were performed with a CM12 Phillips TEM at the Centre Commun de Microscopie Appliquée at the University of Nice-Sophia Antipolis.

For Scanning Electron Microscopy (SEM), soft tissues of microcolonies were removed from the skeleton with 10% NaClO and the skeleton was then rinsed with distilled water. Samples were coated with gold-palladium and observed with a JEOL JSM 6010 LV at the Centre Scientifique de Monaco.

### Confocal microscopy for observations of biominerals and surrounding cells

To investigate that crystals deposited at the growing edge correspond to crystals that give rise to microprotuberances, soft tissues of microcolonies were removed from the skeleton with 0.1% NaClO and the skeleton was then rinsed with distilled water. Samples were then gently decalcified with EDTA 25 mM and immunolabelling was performed with an antibody raised against the soluble organic matrix (SOM) extracted from the axial skeleton of *C. rubrum* ^21^ at a dilution of 1:1000. Then, samples were washed in blocking buffer for 1 hour before incubation at room temperature with biotinylated anti-rabbit antibody (1:250 dilution, Sigma) as secondary antibody. After rinsing in saturating medium and PBS, samples were incubated for 1 hour with streptavidin-Alexa Fluor 568 (Invitrogen). Imaging of decalcified crystals labelled with antibody was performed using an upright confocal microscope Leica TCS SP5 with a 40x water objective (NA 0.80) using an excitation wavelength of 543 nm and emission capture at 592–615 nm. Z-stacks of 37 optical sections of decalcified crystals were captured in 1 µm steps. The individual images were then merged as a Maximum Intensity Projection, using the software Huygens Essential (16.05, Scientific Volume Imaging).

Imaging of mineralized sheets deposited on the coverslip was performed on microcolonies that had been prepared at least six months previously. A stock solution of calcein (2 g.L^−1^, Sigma) was prepared as previously described^54^ and then diluted to a final concentration of 0.1 g.L^−1^ in filtered seawater (FSW) and buffered at pH 8.1 with NaOH. Microcolonies were incubated in calcein solution for 1 hour, washed with filtered seawater and put back in the aquarium. After 24 hours, microcolonies were observed with a Leica TCS SP5 confocal laser scanning microscope to visualize calcein staining of crystals deposited on the coverslip. Calcein-stained microcolonies were then fixed overnight in 4% paraformaldehyde in S22 buffer at 4 °C under gentle agitation. After fixation, samples were washed in PBS and permeabilized in PBS with 0.3% Triton for 40 min. Microcolonies were then incubated in PBS with 0.3% Triton and Alexa Fluor 546 Phalloidin (2%, Molecular probe), washed in PBS, and incubated overnight in PBS/DAPI (2 µg.mL^−1^, Sigma). The stained microcolonies were stored at 4 °C in Petri dishes filled with PBS.To investigate the organization of cells around sclerites, microcolonies were fixed overnight in 4% paraformaldehyde in S22 buffer at 4 °C under gentle agitation, decalcified for one week using 0.25 M EDTA in calcium-free S22 and rinsed in PBS. Tissues were then cut into pieces with a surgical blade before being gently peeled off the coverslip. Pieces of tissues were permeabilized in PBS containing 0.1% Tween 20, rinsed successively in PBS with 0.3% Triton for 40 min, and in PBS. Image-iT FX signal enhancer reagent (Invitrogen) was applied on the tissues for 30 min at room temperature. Tissues were then rinsed in PBS solution and PBS with 0.1% Tween 20, before incubation for two hours in blocking buffer (2% BSA, 0.2% teleostean gelatin, 0.1% Tween 20, 0.3% Triton, 0.5% donkey serum in 0.05 mol.L^−1^ PBS, pH 7.4). Samples were then incubated during two days at 4 °C in PBS containing Alexa Fluor 546 Phalloidin (2.5%, Molecular probe) and primary antibody raised against the soluble organic matrix (SOM) extracted from the sclerites of *C. rubrum* ^21^, at a dilution of 1:2000. Then, tissues were washed in blocking buffer for 1 hour before incubation overnight at 4 °C with biotinylated anti-rabbit antibody (1:250 dilution, Sigma) as secondary antibody. After rinsing in saturating medium and PBS, tissues were incubated for one hour with streptavidin-Alexa Fluor 633 (Invitrogen). After a final wash in PBS, tissues were incubated in PBS/DAPI (2 µg.mL^−1^, Sigma). Stained tissues were finally mounted on glass microscope slides with Permafluor mounting medium (Thermo Scientific).

Imaging of mineralized sheets, sclerites and surrounding cells was carried out with an inverted confocal microscope Leica TCS SP5 with a 40x oil objective (NA 1.3). Images were acquired sequentially for each fluorochrome to avoid crosstalk. To obtain high-resolution images of sclerites cellular environment, confocal microscopy ideal sampling was calculated using the Nyquist calculator (Scientific Volume Imaging). Resulting images were then processed using Huygens Essential software (16.05; Scientific Volume Imaging) to improve the resolution by deconvolution. Maximum Intensity Projection (MIP) was also used to create 3D reconstructions of one sclerite with its surrounding scleroblasts.

### Intra- and extra-cellular pH calibrations

Intracellular and extracellular pH were determined by ratiometric analysis of SNARF-1 (Molecular Probes) by confocal microscopy using two separate calibrations based on protocols developed previously for *S. pistillata* ^13, 38^. Extracellular pH (pHe) calibration was performed by determining the ratio of SNARF-1 fluorescence in filtered seawater adjusted to the range of pH 7–9 containing 45 µM SNARF-1^13, 45^. The pH of seawater calibration solutions (total scale pH) was determined by spectrophotometric analysis of M-cresol purple indicator dye (Acros 199250050)^60^. Intracellular pH (pHi) calibration was performed with the cell permeant acetoxymethyl ester acetate of SNARF-1 (SNARF-1 AM)^38^ in isolated cells of *C. rubrum*. For this purpose, 1 cm long fragments of *C. rubrum* branches were cut with a bone cutter and tissues were removed from the skeleton. The cells were obtained by scraping the tissue that had previously faced the skeleton with a stiff brush into 2 mL of FSW. The cell suspension was then centrifuged (300 g, 3 min, 19 °C), the supernatant removed and cells resuspended in 20 µM SNARF-1 AM in FSW (pH 8, 0.01% pluronic acid F-127, 0.1% DMSO). The suspension of SNARF-1 AM-loaded cells was then transferred into a POC cell cultivation chamber (PECON) and cells were allowed to settle on the glass coverslip. After allowing 20 min for cells to load with SNARF-1 AM, the solution overlying the cells was removed by pipetting and replaced with calibration solutions^61, 62^ (Supplementary Material) ranging from pH 6 to 8.5.

Calibration curves of pHi and pHe are provided in Supplementary data Fig. S1. All calibrations were performed at 19 °C under dark conditions (same conditions as in aquaria). In each calibration the 585/640 nm SNARF-1 fluorescence intensity ratio (R) was related to pH by the following equation:

$${\rm{pH}}={\rm{pKA}}-\mathrm{log}\,[{\rm{R}}-{{\rm{R}}}_{{\rm{B}}}{/{\rm{R}}}_{{\rm{A}}}-{\rm{R}}\times {{\rm{F}}}_{{\rm{B}}}(\lambda )/\text{FA}(\lambda )]$$

where F is fluorescence intensity measured at 640 nm (λ2) and A and B represent the limiting values at the acidic (i.e. pH 6) and basic (i.e. pH 8.5/9) end points of the titration respectively. Ratio (R) was measured in digital regions of interest (ROIs).

### *In vivo* pHi and pHe measurements in microcolonies

Different incubations of microcolonies were necessary for measuring pHi and pHe. Microcolonies growing on coverslips were fitted in a chamber (PeCon) placed on a temperature-controlled microscope stage (Temperable Insert P, PeCon) maintained in darkness at 19 °C, and incubated with free SNARF-1 for pHe measurements, or SNARF-1 AM for pHi measurements. The term “intracellular pH” refers to the pH of the intracellular compartment (cytosol) of calcifying cells and endodermal cells forming gastrodermal canals. “Extracellular pH” includes pH measurements of surrounding seawater and the pH of all extracellular media such as fluids between calcifying cells and biominerals (extracellular calcifying medium), and the fluid flowing in the gastrodermal canals.

For pHi measurements, microcolonies were incubated in 20 µM SNARF-1 AM for 30 min to allow incorporation of probe in tissues, followed by incubation in FSW to remove excess of probe. For pHe measurements, microcolonies were incubated for one hour in a 90 µM SNARF-1 solution, which was replaced with a new SNARF-1 solution to start measurements. Longer incubations were thus necessary to obtain a measurable fluorescence intensity in extracellular media compared to the protocol applied for *S. pistillata* ^13^. Incubation solutions were regularly homogenized by pipetting. Probe concentrations for *in vivo* pHi and pHe measurements were higher than those used for calibrations, but SNARF-1 fluorescence intensity is independent of probe concentration^63, 64^.

Confocal analyses were conducted using a Leica TCS SP5 inverted confocal microscope. SNARF-1 and SNARF-1 AM were excited at 543 nm with a laser power at 50%, and fluorescence emission was captured in the two channels, 585 ± 10 nm and 640 ± 10 nm. For each measurement, the intensity of fluorescence was recorded through the cell or the extracellular medium (Z-stack acquisition with 0.5–1 µm steps) in a region of interest (ROI). The pH was then determined following the equation (see above) where R represents the 585/640 nm fluorescence intensity ratio (R) measured in a ROI. pHi and pHe values are expressed on the NBS scale (National Bureau of Standards) and Total scale respectively.

### Statistical analyses

Statistical analyses were performed with SPSS statistical software. Data were checked for normality using the Shapiro-Wilk’s test and for homoscedasticity of variances using Levene’s test. One-way ANOVAs with Student-Newman-Keuls post hoc analyses were performed on (1) pHi and pHe data for each intra- or extra-cellular compartment, and (2) the size of isolated sclerites according to their color. When conditions were not fulfilled, the non-parametric Kruskal-Wallis test was used.

### Data availability

All the data analyzed in this study are available as supplementary file (Data.xls).

## Electronic supplementary material

Supplementary information

Supplementary dataset

## References

[1] Park E (2012). Estimation of divergence times in cnidarian evolution based on mitochondrial protein-coding genes and the fossil record. Mol. Phylogenet. Evol..

[2] Vielzeuf D, Garrabou J, Baronnet A, Grauby O, Marschal C (2008). Nano to macroscale biomineral architecture of red coral (Corallium rubrum). Am. Mineral..

[3] Tambutté S (2011). Coral biomineralization: From the gene to the environment. J. Exp. Mar. Bio. Ecol..

[4] McCulloch M, Falter J, Trotter J, Montagna P (2012). Coral resilience to ocean acidification and global warming through pH up-regulation. Nat. Clim. Chang..

[5] Venn AA (2013). Impact of seawater acidification on pH at the tissue-skeleton interface and calcification in reef corals. Proc. Natl. Acad. Sci. USA.

[6] Anagnostou E, Huang KF, You CF, Sikes EL, Sherrell RM (2012). Evaluation of boron isotope ratio as a pH proxy in the deep sea coral Desmophyllum dianthus: Evidence of physiological pH adjustment. Earth Planet. Sci. Lett..

[7] Holcomb M (2014). Coral calcifying fluid pH dictates response to ocean acidification. Sci. Rep..

[8] Trotter J (2011). Quantifying the pH ‘vital effect’ in the temperate zooxanthellate coral Cladocora caespitosa: Validation of the boron seawater pH proxy. Earth Planet. Sci. Lett..

[9] Al-Horani FA, Al-Moghrabi SM, De Beer D (2003). The mechanism of calcification and its relation to photosynthesis and respiration in the scleractinian coral Galaxea fascicularis. Mar. Biol..

[10] Ries JB (2011). A physicochemical framework for interpreting the biological calcification response to CO2-induced ocean acidification. Geochim. Cosmochim. Acta.

[11] Cai W-J (2016). Microelectrode characterization of coral daytime interior pH and carbonate chemistry. Nat. Commun..

[12] Tambutté E (2015). Morphological plasticity of the coral skeleton under CO2-driven seawater acidification. Nat. Commun..

[13] Venn AA, Tambutté E, Holcomb M, Allemand D, Tambutté S (2011). Live tissue imaging shows reef corals elevate pH under their calcifying tissue relative to seawater. PLoS One.

[14] McCulloch M (2012). Resilience of cold-water scleractinian corals to ocean acidification: Boron isotopic systematics of pH and saturation state up-regulation. Geochim. Cosmochim. Acta.

[15] Muscatine L, Tambutté É, Allemand D (1997). Morphology of coral desmocytes, cells that anchor the calicoblastic epithelium to the skeleton. Coral Reefs.

[16] Ohno Y (2017). An aposymbiotic primary coral polyp counteracts acidification by active pH regulation. Sci. Rep..

[17] Allemand D (1993). Biology and skeletogenesis of the Mediterranean red coral. Precious coral octocoral Res..

[18] Morel, J.-P., Rondi-Costanzo, C. & Ugolini, D. *Corallo di ieri, corallo di oggi*. (Edipuglia, 2000).

[19] Debreuil J (2012). Molecular cloning and characterization of first organic matrix protein from sclerites of red coral, Corallium rubrum. J. Biol. Chem..

[20] Grillo M-C, Goldberg WM, Allemand D (1993). Skeleton and sclerite formation in the precious red coral Corallium rubrum. Mar. Biol..

[21] Debreuil J (2011). Comparative analysis of the soluble organic matrix of axial skeleton and sclerites of Corallium rubrum: Insights for biomineralization. Comp. Biochem. Physiol. Part B Biochem. Mol. Biol..

[22] Gallmetzer I, Haselmair A, Velimirov B (2010). Slow growth and early sexual maturity: Bane and boon for the red coral Corallium rubrum. Estuar. Coast. Shelf Sci..

[23] Garrabou J, Harmelin JG (2002). A 20-year study on life-history traits of a harvested long-lived temperate coral in the NW Mediterranean: Insights into conservation and management needs. J. Anim. Ecol..

[24] Marschal C, Garrabou J, Harmelin JG, Pichon M (2004). A new method for measuring growth and age in the precious red coral Corallium rubrum (L.). Coral Reefs.

[25] Vielzeuf D (2013). Distribution of sulphur and magnesium in the red coral. Chem. Geol..

[26] Abbiati M, Buffoni G, Caforio G, Di Cola G, Santangelo G (1992). Harvesting, predation and competition effects on a red coral population. Netherlands J. Sea Res..

[27] Garcia-Rodriguez M, Masso C (1986). Estudio biometrico de poblaciones de coral rojo del litoral de Gerona. Biol. Inst. Esp. Ocean..

[28] Harmelin JG (1983). Biologie du corail rouge. Paramètres de populations, croissance et mortalité naturelle. Etat des connaissances en France. FAO Fish Rep.

[29] Bourne GC (1899). Studies on the structure and formation of the calcareous skeleton of the Anthozoa. Q. J. Microsc. Sci..

[30] Woodland W (1905). Studies in spicule formation. II— Spicule formation in Alcyonium digitatum; with remarks on the histology. Q. J. Microsc..

[31] Kingsley RJ, Watabe N (1982). Ultrastructural investigation of spicule formation in the gorgonian Leptogorgia virgulata (Lamarck) (Coelenterata: Gorgonacea). Cell Tissue Res..

[32] Kingsley RJ (1984). Spicule formation in the invertebrates with special reference to the Gorgonian Leptogorgia virgulata. Am. Zool..

[33] Kingsley RJ, Bernhardt AM, Wilbur KM, Watabe N (1987). Scleroblast cultures from the Gorgonian Leptogorgia virgulata (Lamarck). Vitr. Cell. Dev. Biol..

[34] Dunkelberger DG, Watabe N (1974). An ultrastructural study on spicule formation in the pennatulid colony Renilla reniformis. Tissue Cell.

[35] Goldberg WM, Benayahu Y (1987). Spicule formation in the Gorgonian coral Pseudoplexaura flagellosa. 1:Demonstration of intracellular and extracellular growth and the effect of ruthenium red during decalcification. Bull. Mar. Sci..

[36] Jeng MS, Huang HD, Dai CF, Hsiao YC, Benayahu Y (2011). Sclerite calcification and reef-building in the fleshy octocoral genus Sinularia (Octocorallia: Alcyonacea). Coral Reefs.

[37] Menzel LP, Tondo C, Stein B, Bigger CH (2015). Histology and ultrastructure of the coenenchyme of the octocoral Swiftia exserta, a model organism for innate immunity/graft rejection. Zoology.

[38] Venn AA (2009). Imaging intracellular pH in a reef coral and symbiotic anemone. Proc. Natl. Acad. Sci. USA.

[39] Laurent J, Tambutté S, Tambutté É, Allemand D, Venn AA (2013). The influence of photosynthesis on host intracellular pH in scleractinian corals. J. Exp. Biol..

[40] Gibbin EM, Putnam HM, Davy SK, Gates RD (2014). Intracellular pH and its response to CO2-driven seawater acidification in symbiotic versus non-symbiotic coral cells. J. Exp. Biol..

[41] Le Goff C (2016). Carbonic anhydrases in cnidarians: Novel perspectives from the octocorallian Corallium rubrum. PLoS One.

[42] Al-Horani FA, Al-Moghrabi SM, De Beer D (2003). Microsensor study of photosynthesis and calcification in the scleractinian coral, Galaxea fascicularis: Active internal carbon cycle. J. Exp. Mar. Bio. Ecol..

[43] Zoccola D (2004). Molecular cloning and localization of a PMCA P-type calcium ATPase from the coral Stylophora pistillata. Biochim. Biophys. Acta.

[44] Lowenstam, H. A. & Weiner, S. *On biomineralization*. (Oxford University Press, 1989).

[45] Bentov S, Brownlee C, Erez J (2009). The role of seawater endocytosis in the biomineralization process in calcareous foraminifera. Proc. Natl. Acad. Sci. USA.

[46] de Nooijer LJ, Langer G, Nehrke G, Bijma J (2009). Physiological controls on seawater uptake and calcification in the benthic foraminifer Ammonia tepida. Biogeosciences.

[47] Rink S, Kühl M, Bijma J, Spero HJ (1998). Microsensor studies of photosynthesis and respiration in the symbiotic foraminifer Orbulina universa. Mar. Biol..

[48] Anning T, Nimer N, Merrett MJ, Brownlee C (1996). Costs and benefits of calcification in coccolithophorids. J. Mar. Syst..

[49] Borowitzka MA, Larkum AWD (1987). Calcification in algae: Mechanisms and the role of metabolism. CRC. Crit. Rev. Plant Sci..

[50] McConnaughey TA, Whelan JF (1997). Calcification generates protons for nutrient and bicarbonate uptake. Earth-Science Rev..

[51] De Beer D, Larkum AWD (2001). Photosynthesis and calcification in the calcifying algae Halimeda discoidea studied with microsensors. Plant, Cell Environ..

[52] Furla P, Galgani I, Durand I, Allemand D (2000). Sources and mechanisms of inorganic carbon transport for coral calcification and photosynthesis. J. Exp. Biol..

[53] Allison N, Cohen I, Finch AA, Erez J, Tudhope AW (2014). Corals concentrate dissolved inorganic carbon to facilitate calcification. Nat. Commun..

[54] Tambutté E (2012). Calcein labelling and electrophysiology: insights on coral tissue permeability and calcification. Proc. R. Soc. B Biol. Sci..

[55] Mann, S. *Biomineralization: Principles ans concepts in bioinorganic materials chemistry*. (New York: Oxford University Press, 2001).

[56] Allemand, D., Mayer‐Gostan, N., De Pontual, H., Boeuf, G. & Payan, P. In *Handbook of Biomineralization: Biological aspects and structure formation*. 291–308 (2007).

[57] Bramanti L (2013). Detrimental effects of ocean acidification on the economically important Mediterranean red coral (Corallium rubrum). Glob. Chang. Biol..

[58] Cerrano C (2013). Red coral extinction risk enhanced by ocean acidification. Sci. Rep..

[59] Tambutté E (2007). Observations of the tissue-skeleton interface in the scleractinian coral Stylophora pistillata. Coral Reefs.

[60] Dickson, A. G., Sabine, C. L. & Christian, J. R. *Guide to best practices for ocean CO 2 measurements*. (2007).

[61] Herrera F, Lopez I, Egea R, Zanders P (1989). Short-term osmotic response of cells and tissues of the sea anemones, Condylactis gigantea. Comp. Biochem. Physiol. A Comp. Physiol..

[62] Goiran C, Allemand D, Galgani I (1997). Transient Na+ stress in symbiotic dinoflagellates after isolation from coral-host cells and subsequent immersion in seawater. Mar. Biol..

[63] Buckler KJ, Vaughan-Jones RD (1990). Application of a new pH-sensitive fluoroprobe (carboxy-SNARF-1) for intracellular pH measurement in small, isolated cells. Eur. J. Physiol..

[64] Seksek O, Henry-Toulmé N, Sureau F, Bolard J (1991). SNARF-1 as an intracellular pH indicator in laser microspectrofluorometry: a critical assessment. Anal. Biochem..

