# Supplementary material for: In vivo pH measurement at the site of calcification in an octocoral
Source: Sci Rep. 2017 Sep 11;7:11210. doi: 10.1038/s41598-017-10348-4 (PMC5593875; doi:10.1038/s41598-017-10348-4)
Supplement: Supplementary file 1 — Supplementary information [file 41598_2017_10348_MOESM1_ESM.doc]

***In vivo* pH measurement at the site of calcification in an octocoral**

Le Goff Carine, Tambutté Eric, Venn Alexander A., Techer Nathalie, Allemand Denis, Tambutté Sylvie*

**1) Supplementary Material and Methods**

**Biological material**

Branches of *Corallium rubrum* (Linnaeus, 1758) were removed from parent colonies at 10 meters depth near Marseille (Plane Island, Gulf of Lion) by IMBE/Marseille. Sampling permission was delivered by the Maritime Prefect of the Bouches-du-Rhône, France. Visual inspection confirmed that healing of the parent colony occurred within two weeks after removal of a branch. Branches were transferred to the Centre Scientifique de Monaco and maintained in an open-circuit seawater aquarium supplied with filtered Mediterranean seawater (18 ± 2°C). Branches were fed every working day with frozen rotifers and twice a week with live *Artemia salina* nauplii.

**Transmission Electron Microscopy for histology of microcolonies**

Samples preparation, fixation and decalcification for Transmission Electron Microscopy (TEM) were performed according to protocols described in Tambutté *et al.* ^1^. Briefly, microcolonies were fixed overnight (0.085 M Sorensen phosphate buffer, 0.5 M sucrose, 4% glutaraldehyde, pH 7.8, 4°C) and then transferred in a solution for decalcification (0.085 M Sorensen phosphate buffer, 0.5 M sucrose, 2% glutaraldehyde, 0.5 M EDTA, pH 7.8, 4°C). Samples were then rinsed in Sorensen phosphate buffer, post-fixed for 1 h with 1% osmium tetroxide in Sorensen phosphate buffer, dehydrated through a graded series of ethanol and embedded in Epon resin. Observations of 1 µm thick sections were performed with a CM12 Phillips TEM at the Centre Commun de Microscopie Appliquée at the University of Nice-Sophia Antipolis.

**pH calibration solutions**

Calibration solutions contained 60 mM Na^+^, 200 mM K^+^, 190 mM Cl^-^, 25 mM Pipes (pH 6-7.5) or Tricine (pH 8–8.5, with mannitol added to adjust osmolarity to 1100 mosm.L^-1^) ^2,3^. The ionophore nigericin was included in each calibration solution at a concentration of 60 µM.

**2) Supplementary Tables and Figures**


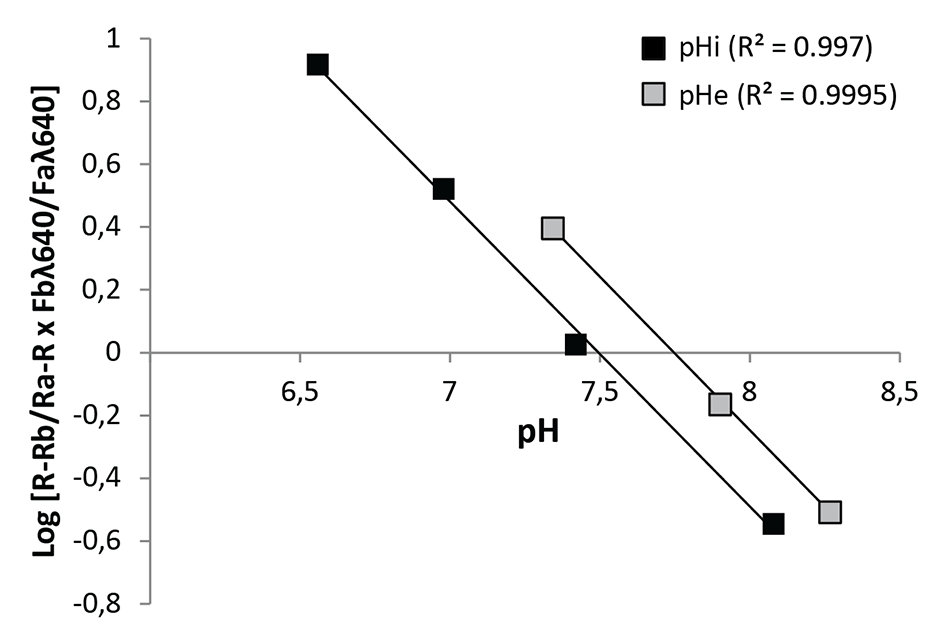


S1 Fig. Calibration of intracellular and extracellular pH. Calibration of intracellular (pHi) and extracellular (pHe) pH with the ratio of SNARF-1 AM and SNARF-1 fluorescence at 585 and 640 nm. See Material and Methods for details.


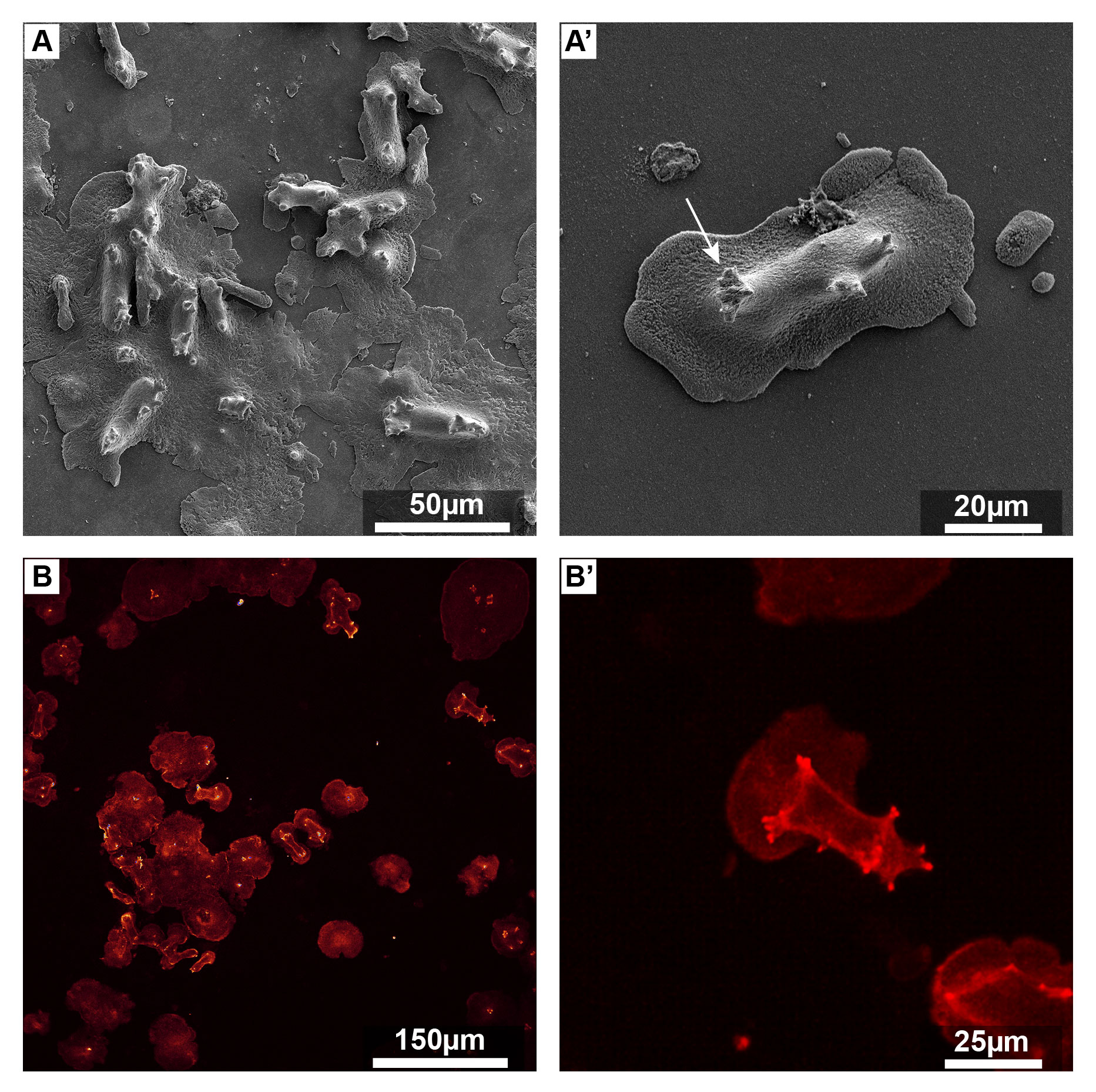


S2 Fig. **Images showing that the dumbbell-shaped crystals deposited at the growing edge give rise to microprotuberances.** (**A-A’**). Scanning electron micrograph at the growing edge showing that dumbbell-shaped crystals give rise to microprotuberances (arrow). (**B-B’**). Decalcified dumbbell-shaped crystals and mineral sheet labelled with an antibody against organic matrix showing intense labelling of the microprotuberances that point outwards from the dumbbell-shaped crystals. Maximum intensity projection of optical sections acquired with a confocal microscope.


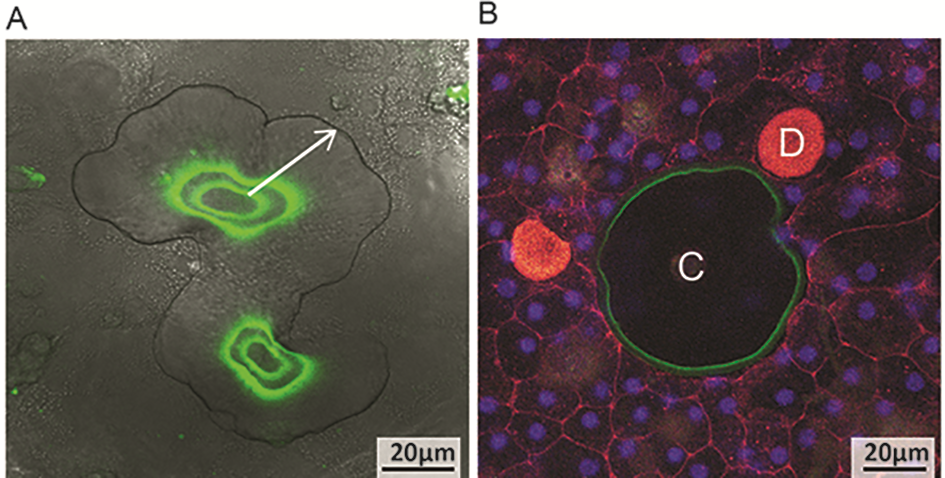


S3 Fig. Confocal images of calcein labeling of mineral sheets deposited on the coverslip and labeling of cells showing crystals-tissue organization. (A) Confocal image merged with transmitted light image of mineral sheets stained with calcein (green) at two days interval and observed nine days after the first labeling. Lateral extension of the mineral sheet between the 1^st^ and 9^th^ day is denoted by the white arrow. (B) Rounded calcified sheet (C) marked with calcein (green) surrounded by cells labeled with Phalloidin (red) and DAPI (blue). D: Desmocytes.


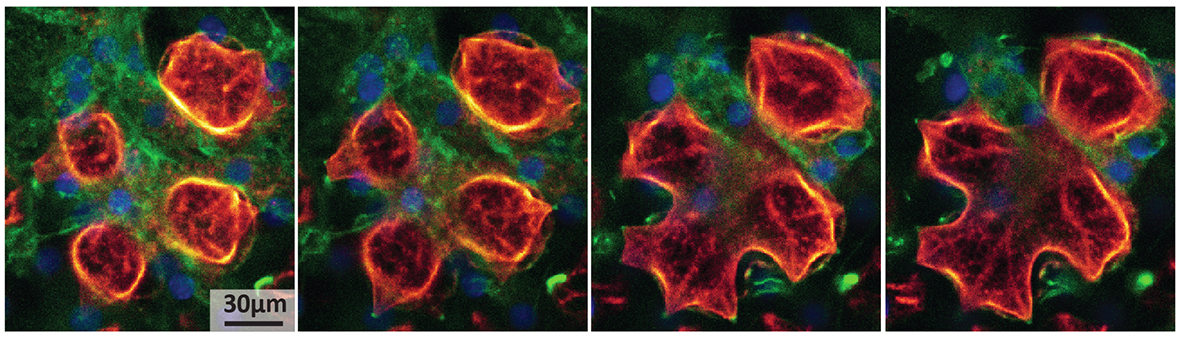


S4 Fig. Optical sections acquired with a confocal microscope through a mature sclerite labeled with anti sclerite SOM (red), and cells labeled with Phalloidin (actin, green) and DAPI (nuclei, blue). Mature sclerites are present in the mesoglea where they have been extruded from secondary scleroblasts with a cell network passing between protuberances.


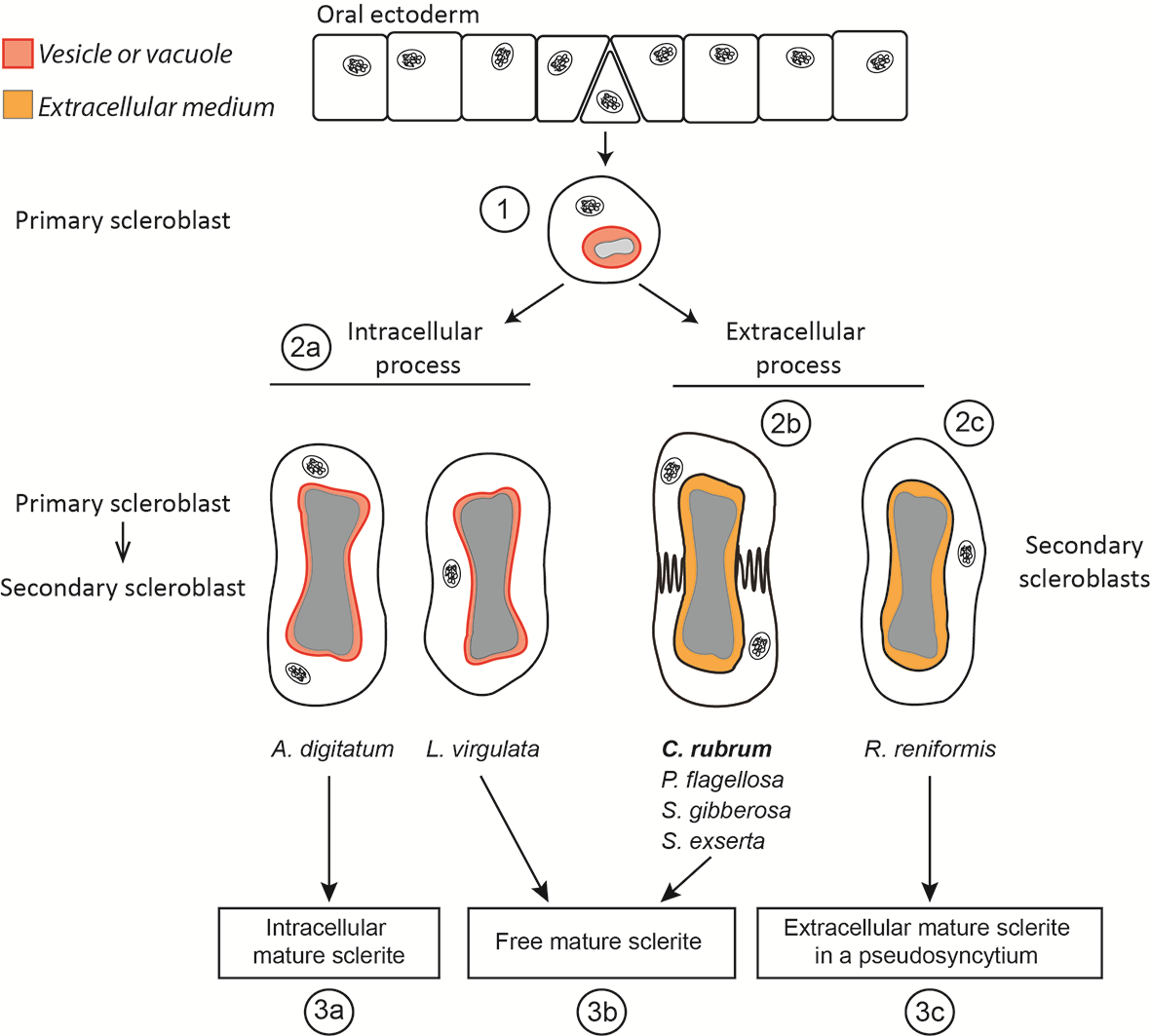


S5 Fig. Summary of cellular processes of sclerites formation in the Octocorallians. The interstitial cells of the oral ectoderm give rise to scleroblasts as suggested by Dunkelberger & Watabe, 1974 and Woodland, 1905. Two distinct functional forms of scleroblasts have been highlighted: primary and secondary scleroblasts. The initiation of calcification would take place within a vesicle or vacuole of a primary scleroblast (1). The growth of sclerite can continue intracellularly (2a) either within a syncytium formed by incomplete cytokinesis (*Alcyonium digitatum*) or within the scleroblast vacuole (*Leptogorgia virgulata*). The sclerite formation can involve an extracellular medium formed following the aggregation of secondary scleroblasts (2b ; *Corallium rubrum*, *Pseudoplexora flagellosa*, *Sinularia gibberosa*, *Swiftia exerta*) or following the formation of a pseudosyncytium by fusion of plasma membranes of secondary scleroblasts (2c ; *Renilla reniformis*). Finally, steps (3a-b-c) refere to mature sclerites.

See the main manuscript for references 29-37.

**S1 Table. Summary of studies regarding the use of microcolonies of scleractinian corals growing on coverslips or glass slides.** Stars (*) indicate studies related to pH measurements at site of calcification.

| **Authors** | **Year** | **Publication title** |
| --- | --- | --- |
| * Tambutté et al. | 1996 ^4^ | Processus de calcification d'un scléractiniaire hermatypique *Stylophora pistillata* (esper, 1797). Etude de la croissance *in situ* sur l'atoll de mururoa. |
| * Muscatine et al. | 1997 | Morphology of coral desmocytes, cells that anchor the calicoblastic epithelium to the skeleton. |
| * Reynaud-Vaganay et al. | 1999 ^5^ | A novel culture technique for scleractinian corals: Application to investigate changes in skeletal δ^18^O as a function of temperature. |
| Shafir et al. | 2001 ^6^ | Nubbing of coral colonies: A novel approach for the development of inland broodstocks. |
| Shafir et al. | 2003 ^7^ | The use of coral nubbins in coral reef ecotoxicology testing |
| * Puverel et al. | 2005 ^8^ | Antibodies against the organic matrix in scleractinians: A new tool to study coral biomineralization. |
| Shafir et al. | 2006 ^9^ | Coral nubbins as source material for coral biological research: A prospectus. |
| * Raz-Bahat et al. | 2006 ^10^ | *In vivo* light-microscopic documentation for primary calcification processes in the hermatypic coral *Stylophora pistillata*. |
| * Tambutté et al. | 2007 | Observations of the tissue-skeleton interface in the scleractinian coral *Stylophora pistillata*. |
| * Venn et al. | 2011 | Live tissue imaging shows reef corals elevate pH under their calcifying tissue relative to seawater. |
| * Tambutté et al. | 2012 | Calcein labeling and electrophysiology: insights on coral tissue permeability and calcification. |
| * Venn et al. | 2013 | Impact of seawater acidification on pH at the tissue-skeleton interface and calcification in reef corals. |
| * Holcomb et al. | 2014 | Coral calcifying fluid pH dictates response to ocean acidification. |
| * Tambutté et al. | 2015 | Morphological plasticity of the coral skeleton under CO_2_-driven seawater acidification. |
| * Shapiro et al. | 2016 ^11^ | A coral-on-a-chip microfluidic platform enabling live-imaging microscopy of reef-building corals. |
| Ohno et al. | 2017 | An aposymbiotic primary coral polyp counteracts acidification by active pH regulation |


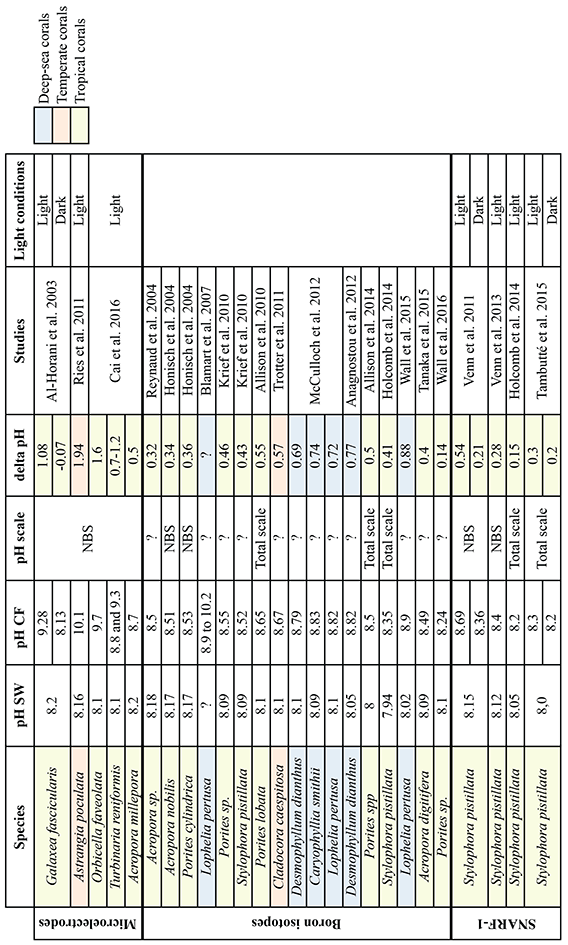
**S2 Table. Summary of studies regarding the pH at the site of calcification of scleractinian corals (Hexacorallia) compared to the surrounding seawater pH.** pH SW: pH of the surrounding seawater ; pH CF : pH of the calcifying fluid.

1. **Supplementary References**

1. Tambutté, E. *et al.* Observations of the tissue-skeleton interface in the scleractinian coral Stylophora pistillata. *Coral Reefs* **26,** 517–529 (2007).

2. Herrera, F., Lopez, I., Egea, R. & Zanders, P. Short-term osmotic response of cells and tissues of the sea anemones, Condylactis gigantea. *Comp. Biochem. Physiol. A Comp. Physiol.* **92,** 377–384 (1989).

3. Goiran, C., Allemand, D. & Galgani, I. Transient Na+ stress in symbiotic dinoflagellates after isolation from coral-host cells and subsequent immersion in seawater. *Mar. Biol.* **129,** 581–589 (1997).

4. Tambutté, É., Allemand, D., Mueller, E. & Jaubert, J. A compartmental approach to the mechanism of calcification in hermatypic corals. *J. Exp. Biol.* **199,** 1029–1041 (1996).

5. Reynaud-Vaganay, S., Gattuso, J. P., Cuif, J. P., Jaubert, J. & Juillet-Leclerc, A. A novel culture technique for scleractinian corals: Application to investigate changes in skeletal δ18O as a function of temperature. *Mar. Ecol. Prog. Ser.* **180,** 121–130 (1999).

6. Shafir, S., Van Rijn, J. & Rinkevich, B. Nubbing of coral colonies: A novel approach for the development of inland broodstocks. *Aquarium Sci. Conserv.* **3,** 183–190 (2001).

7. Shafir, S., Van Rijn, J. & Rinkevich, B. The use of coral nubbins in coral reef ecotoxicology testing. *Biomol. Eng.* **20,** 401–406 (2003).

8. Puverel, S. *et al.* Antibodies against the organic matrix in scleractinians: A new tool to study coral biomineralization. *Coral Reefs* **24,** 149–156 (2005).

9. Raz-Bahat, M., Erez, J. & Rinkevich, B. In vivo light-microscopic documentation for primary calcification processes in the hermatypic coral Stylophora pistillata. *Cell Tissue Res.* **325,** 361–368 (2006).

10. Shafir, S., Van Rijn, J. & Rinkevich, B. Coral nubbins as source material for coral biological research: A prospectus. *Aquaculture* **259,** 444–448 (2006).

11. Shapiro, O. H., Kramarsky-Winter, E., Gavish, A. R., Stocker, R. & Vardi, A. A coral-on-a-chip microfluidic platform enabling live-imaging microscopy of reef-building corals. *Nat. Commun.* **7,** 10860 (2016).

12. Reynaud, S., Hemming, N. G., Juillet-Leclerc, A. & Gattuso, J. P. Effect of pCO2 and temperature on the boron isotopic composition of the zooxanthellate coral Acropora sp. *Coral Reefs* **23,** 539–546 (2004).

13. Hönisch, B. *et al.* Assessing scleractinian corals as recorders for paleo-pH: Empirical calibration and vital effects. *Geochim. Cosmochim. Acta* **68,** 3675–3685 (2004).

14. Blamart, D. *et al.* Correlation of boron isotopic composition with ultrastructure in the deep-sea coral Lophelia pertusa: Implications for biomineralization and paleo-pH. *Geochemistry, Geophys. Geosystems* **8,** 1–11 (2007).

15. Krief, S. *et al.* Physiological and isotopic responses of scleractinian corals to ocean acidification. *Geochim. Cosmochim. Acta* **74,** 4988–5001 (2010).

16. Allison, N., Finch, A. a. & EIMF. 11B, Sr, Mg and B in a modern Porites coral: the relationship between calcification site pH and skeletal chemistry. *Geochim. Cosmochim. Acta* **74,** 1790–1800 (2010).

17. McCulloch, M. *et al.* Resilience of cold-water scleractinian corals to ocean acidification: Boron isotopic systematics of pH and saturation state up-regulation. *Geochim. Cosmochim. Acta* **87,** 21–34 (2012).

18. Wall, M., Ragazzola, F., Foster, L. C., Form, A. & Schmidt, D. N. Enhanced pH up-regulation enables the cold-water coral Lophelia pertusa to sustain growth in aragonite undersaturated conditions. *Biogeosciences Discuss.* **12,** 6757–6781 (2015).

19. Wall, M. *et al.* Internal pH regulation facilitates in situ long-term acclimation of massive corals to end-of-century carbon dioxide conditions. *Sci. Rep.* **6,** 30688 (2016).

20. Tanaka, K. *et al.* Response of Acropora digitifera to ocean acidification: constraints from δ11B, Sr, Mg, and Ba compositions of aragonitic skeletons cultured under variable seawater pH. *Coral Reefs* **34,** 1139–1149 (2015).
